# Fixed-bed adsorption of Pb(ii) and Cu(ii) from multi-metal aqueous systems onto cellulose-*g*-hydroxyapatite granules: optimization using response surface methodology†

**DOI:** 10.1039/d3ra04974d

**Published:** 2023-11-01

**Authors:** Salah Eddine Marrane, Karim Dânoun, Youness Essamlali, Soumia Aboulhrouz, Said Sair, Othmane Amadine, Ilham Jioui, Abdallah Rhihil, Mohamed Zahouily

**Affiliations:** a Laboratory of Materials, Catalysis & Valorization of Natural Resources, Hassan II University FST-Mohammedia Morocco m.zahouily@mascir.ma; b Moroccan Foundation for Advanced Science, Innovation and Research (MAScIR) Benguerir Morocco; c Mohammed VI Polytechnic University Lot 660-Hay Moulay Rachid Benguerir Morocco karim.DANOUN@um6p.ma

## Abstract

We prepared cellulose microfibrils-*g*-hydroxyapatite (CMFs-*g*-HAP_N_ (8%)) in a granular form. We evaluated the ability of these granules to eliminate Pb(ii) and Cu(ii) ions from aqueous solution in dynamic mode using a fixed-bed adsorption column. Several operating parameters (inlet ion concentration, feed flow rate, bed height) were optimized using response surface methodology (RSM) based on a Doehlert design. Based on ANOVA and regression analyses, adsorption was found to follow the quadratic polynomial model with *p* < 0.005, *R*^2^ = 0.976, and *R*^2^ = 0.990, respectively, for Pb(ii) and Cu(ii) ions. Moreover, three kinetic models (Adams–Bohart, Thomas, Yoon–Nelson) were applied to fit our experimental data. The Thomas model and Yoon–Nelson model represented appropriately the whole breakthrough curves. The Adams–Bohart model was suitable only for fitting the initial part of the same curves. Our adsorbent exhibited high selectivity towards Pb(ii) over Cu(ii) ions in the binary metal system, with a maximum predicted adsorption capacity of 59.59 ± 3.37 and 35.66 ± 1.34 mg g^−1^, respectively. Under optimal conditions, multi-cycle sorption–desorption experiments indicated that the prepared adsorbent could be regenerated and reused up to four successive cycles. The prepared CMFs-*g*-HAP_N_ was an efficient and effective reusable adsorbent for removal of heavy metals from aqueous systems, and could be a suitable candidate for wastewater treatment on a large scale.

## Abstract 2

Prepared CMFs-*g*-HAP_N_ was an efficient and effective reusable adsorbent for removal of heavy metals from aqueous systems, and could be a suitable candidate for wastewater treatment on a large scale.

## Graphical abstract

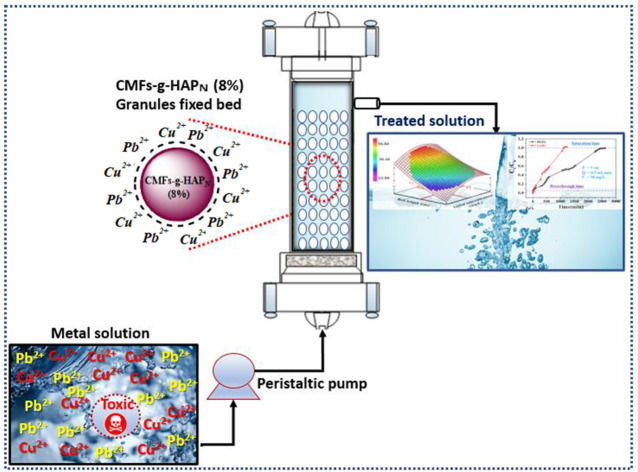

## 1. Introduction

Water pollution by different heavy metals ions has become a serious environmental problem worldwide owing to the high toxicity of these elements, which can cause a serious risk to aquatic ecosystems and humans even at a low concentration.^1^ Among all heavy metals, particular interest has been given to Pb(ii) and Cu(ii) ions in view of their indispensability and to their application in various industrial activities (*e.g.*, electroplating, metal-finishing, textile, battery storage). These features enable generation of an enormous amount of untreated wastewater with various concentrations of these contaminants that are finally discharged into natural water bodies (oceans, rivers, lakes).^2^ For these reasons, intensive effort has been undertaken during the last few decades to control the concentration of these hazardous metal species and to establish strict regulations before their discharge into receiving bodies. According to the US Environmental Protection Agency, the maximum allowable limits for Pb(ii) and Cu(ii) in discharged wastewater are set at 1.3 and 0.05 ppm, respectively.^3^ Considering these issues, an effective research effort has been carried out during recent decades for developing alternative processes to reduce the level of toxic metals in wastewater. Conventional methods such as chemical precipitation, coagulation and flocculation, hybrid electrocoagulation membranes, reverse osmosis, and ion exchange face numerous constraints (*e.g.*, technical efficiency, environmental impact, economic competitiveness) that limit their utilization at the industrial level.^4–7^ However, the adsorption process has received attention (particularly for the treatment of dilute effluents) to provide a good compromise between competitiveness and efficiency. Different materials have been used as an adsorbent for the removal of heavy metal ions from contaminated water. Forms of activated carbon (powdered or granular form) are widely used as commercial adsorbents in view of their microporous structure and large specific surface area.^8^ Nevertheless, the high cost associated with the utilization and reuse of commercial forms of activated carbon in the adsorption process has led many scholars to search for more effective and economic alternative adsorbents. More recently, low-cost hybrid adsorbents originating from abundant natural resources have become among the most sustainable novel materials for elimination of heavy metals. In this regard, Moroccan natural phosphate and date-palm waste were selected as alternative and low-cost raw materials to prepare cellulose microfibrils-grafted-hydroxyapatite (CMFs-*g*-HAP_N_). This hybrid material was shown in our previous work to be an efficient and potential adsorbent in powder form for the uptake of Pb(ii) and Cu(ii) from aqueous solution with a maximum adsorption capacity of 143.80 mg g^−1^ and 79.05 mg g^−1^, respectively in a batch process.^9^ However, the batch mode was mainly limited to the treatment of small-scale wastewater, and less convenient for the industrial scale. Hence, much attention has been given to dynamic adsorption involving a fixed-bed column for the removal of various contaminants from aqueous media. Compared with batch mode, adsorption in column systems is thought to be very helpful due its industrial-scale applicability, and can treat large volumes of wastewater with a high degree of accuracy. To evaluate the performance of adsorption in continuous fixed-bed operations, it is necessary to examine various operating conditions, such as bed depth, linear flow rate through the bed, and inlet solute concentration. The conventional method used to describe the influence of all these factors requires many experimental runs and is therefore time-consuming, which makes it ineffective for some rigorous studies.^9,10^ To overcome these limitations, many researchers have been directed towards the exploitation of statistical experimental designs involving response surface methodology (RSM). These are very effective tools in the design of tests, the study of intra-factor effects, as well as the modelling of physicochemical processes and their optimization.^11^ Among the different matrix designs, the Doehlert Design (DD) has been widely applied in the optimization of pollutant adsorption thanks to its flexibility and excellent outcomes.^12,13^ In the light of these insights and in continuation of our earlier works in investigating interesting low-cost materials for wastewater treatment,^14–22^ we investigated the applicability of CMFs-*g*-HAP_N_ (8%) granules for the removal of Pb(ii) and Cu(ii) ions from aqueous solution in a continuous process. Application of these granules for the removal of heavy metals from wastewater effluents in continuous mode using fixed-bed columns has not been reported previously. Important parameters of the design process, such as inlet metal-ion concentration, flow rate of fluid, and column-bed height were investigated using the DD. Moreover, the correlation of the empirical data to several models (Thomas, Bohart–Adams, Yoon–Nelson) were analysed to explain breakthrough curves.

## 2. Experimental

### 2.1. Materials and methods

The phosphate rock used in the present study was provided by the OCP group (a leader in the phosphate industry in the Moroccan Kingdom). Cellulose microfibrils were obtained from the petiole wood from date palm originating in the Errachidia region of Morocco. The chemical reagents used in this study (all from MilliporeSigma) were copper sulfate (CuSO_4_·5H_2_O), lead nitrate (Pb(NO_3_)_2_), sodium hydroxide (NaOH), sodium hypochlorite (NaClO), hydrochloric acid (HCl), acetic acid (CH_3_COOH), sulfuric acid (H_2_SHO_4_), calcium chloride (CaCl_2_) and ethylenediaminetetraacetic acid disodium salt dihydrate (EDTA-Na_2_). All chemicals were of analytical grade and used without further purification.

### 2.2. Preparation CMFs-*g*-HAP_N_ (8%) granules

CMFs-*g*-HAP_N_ (8%) powder was synthesized *via* the *in situ* wet chemical precipitation method described in our previous study. The detailed preparation process, as well as full characterization of the prepared hybrid adsorbent (using thermogravimetric analysis (TGA), Fourier transform infrared (FTIR) spectroscopy, X-ray diffraction (XRD) analysis, ^31^P-nuclear magnetic resonance (NMR) spectroscopy, scanning electron microscopy (SEM), and Brunauer–Emmett–Teller (BET) calculations) are also given in our previous work.^9^ CMFs-*g*-HAP_N_ (8%) granules were prepared in a small-scale rolling-drum granulator *via* a wet-granulation process. Briefly, a predefined amount of CMFs-*g*-HAP_N_ (8%) powder dried and ground (120–150 μm in size) was introduced into a granulator bowl. This step was followed by a wet-granulation procedure which consisted of periodically spraying water vapor to favor the formation of solid bridges between wet grains under a constant rotation speed (30 rpm) as well as controlling the size of formed granules. After a granulation time of 30 min, the obtained granules were collected, sieved manually, and an abundant fraction of granules (2–4 mm in size) were collected and spread out on a tray for drying at 105 °C for 24 h. Tests of mechanical strength and the orientation of adsorption of samples were evaluated to select suitable granules that could be used in this experiment. Scheme S1 in ESI† illustrates the preparation steps of CMFs-*g*-HAP_N_ (8%) granules.

### 2.3. Study on fixed-bed column adsorption

Experiments on fixed-bed adsorption were carried out using a glass column with an internal diameter of 1.5 cm and length of 25 cm. CMFs-*g*-HAP_N_ (8%) granules (2–4 mm) were packed between layers of stainless-steel filters (0.5 mm) for uniform flow and glass beads to avoid loss of material. Synthetic wastewater of Pb(ii) and Cu(ii) ions was pumped into the column in the up-flow mode with the help of a peristaltic pump (LabV6; Shenchen) at a controlled flow rate. The pH of the inlet effluent was adjusted using HCl (0.01 M) and NaOH (0.01 M). All experiments were carried out at room temperature. Effluent samples were collected from the outlet of the column at regular time intervals. The residual concentration of Pb(ii) and Cu(ii) ions in effluent samples was quantified using an atomic absorption spectrometer with suitable hollow cathode lamps at a wavelength of 283.3 and 324.8 nm, respectively. Operating parameters such as the initial concentration (10–50 mg L^−1^), flow rate (2–11 mL min^−1^), and bed height (3–15 cm) were examined in terms of the effect they had on adsorption. Breakthrough curves were obtained by continuous monitoring of this process. In this respect, the effluent volume *V*_eff_ (mL), total mass of metal adsorbed *q*_total_ (mg) as well as equilibrium metal-ion uptake or adsorption maximum capacity (*q*_e_) (mg g^−1^) can be calculated using eqn (1)–(3):

1 *V*_eff_ = *Q* × *t*_total_

2
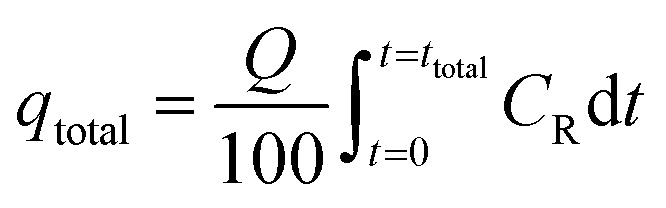
3
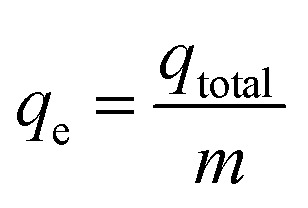
where *t*_total_ is the total flow time (min), *Q* is the volumetric flow rate which circulates through the column (mL min^−1^), *C*_R_ is the concentration of metal removal (mg L^−1^), and *m* is the weight of the granules bed in the column (g).

### 2.4. Optimization of RSM-DD

RSM coupled with the DD (*i.e.*, RSM-DD) was used to evaluate the individual as well as the combined effect of the independent variables that influence the adsorption process of divalent cations Pb(ii) and Cu(ii) onto CMFs-*g*-HAP_N_ (8%) granules. Three independent variables, the initial concentration of the metal (*C*_0_), bed depth of granules in the column (*Z*), and flow rate of the solution (*Q*), were included in RSM-DD measurements. These parameters were selected based on literature reports for heavy-metal adsorption using fixed-bed columns as well as the results of preliminary studies performed by our research team. The coded levels of variables as “low” (−1), “middle” (0), and “high” (+1) as well as their units are presented in Table 1.

**Table 1:** Experimental ranges and levels in the DD for removal of Pb(ii) and Cu(ii)

Factor	Levels		
	Low (−1)	Center (0)	High (+1)
*X*_1_: fixed bed length (*Z*, cm)	3	9	15
*X*_2_: flow rate (*Q*, mL min^−1^)	2	6.5	11
*X*_3_: initial metals concentration (*C*_0_, mg L^−1^)	10	30	50

All data on the experimental design were analysed using NemrodW software and the responses (adsorption capacity) were fitted to a polynomial quadratic model using eqn (4):

4 *Y* = *b*_0_ + ∑*b*_*i*_*X*_*i*_ + ∑*b*_*ii*_*X*_*i*_^2^ + ∑*b*_*ij*_*X*_*i*_*X*_*j*_ + *ε*

where predicted responses are shown by *Y*; variables are marked by *X*_i_ and *X*_*j*_; *b*_0_ is a fixed coefficient; *b*_*j*_, *b*_*jj*_, and *b*_*ij*_ denote the interface coefficient of the linear, quadratic, and second-order terms, respectively; *ε* indicates the model error. Analysis of variance (ANOVA) was executed to ascertain the significance of the regression model. Two-dimensional (2D)–3D surface plots were generated to determine the optimum operating conditions for high removal of heavy metals.

### 2.5. Modeling of breakthrough curves

Various mathematical models have been developed to predict the dynamic behaviour of a fixed-bed column. Thomas,^23–25^ Yoon–Nelson,^26^ and Bohart–Adams^27^ models are the most widely used in dynamic adsorption processes to predict the breakthrough curve. The equations of these models can be expressed as follows:

Thomas:5
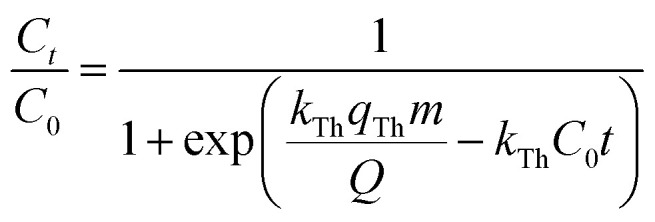


Bohart–Adams:6
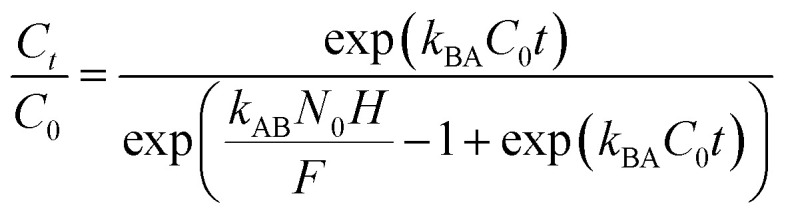


Yoon–Nelson:7
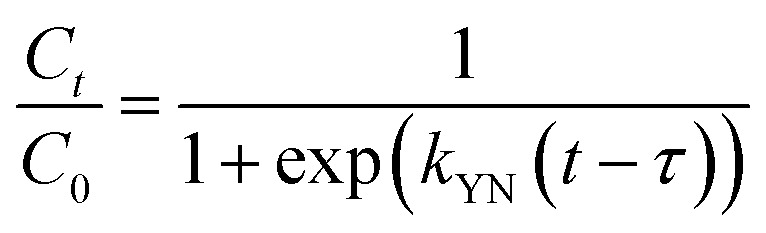
where *k*_Th_ (mL min^−1^ mg^−1^) and *q*_Th_ (mg g^−1^) are the kinetic rate and the adsorption capacity of the Thomas model, respectively. *k*_BA_ (mL min^−1^ mg^−1^) is the kinetic rate of the Bohart–Adams model, *N*_0_ (mg L^−1^) is the saturation concentration, *F* (cm min^−1^) is the linear rate of the solution, *k*_YN_ (min^−1^) is the rate constant of the Yoon–Nelson model, and *τ* (min) is the time taken to reach 50% of *C*_0_.

### 2.6. Study on desorption and regeneration

After adsorption, distilled water was fed through column (*Z* = 6 cm) to remove unadsorbed Pb(ii) and Cu(ii) ions, then followed by 0.001 M EDTA-Na_2_ at a flow rate of 2 mL min^−1^. After the first regeneration, adsorption studies were repeated using identical adsorption conditions. After that, the column was regenerated again and subjected to further adsorption until its capacity was reduced significantly. The effluent was collected at a fixed time interval, and the concentrations of Pb(ii) and Cu(ii) ions in these effluents were determined by atomic absorption spectroscopy (AAS).

## 3. Results and discussion

### 3.1. Selection of suitable adsorbent granules and characterization

**Fig. 1:**
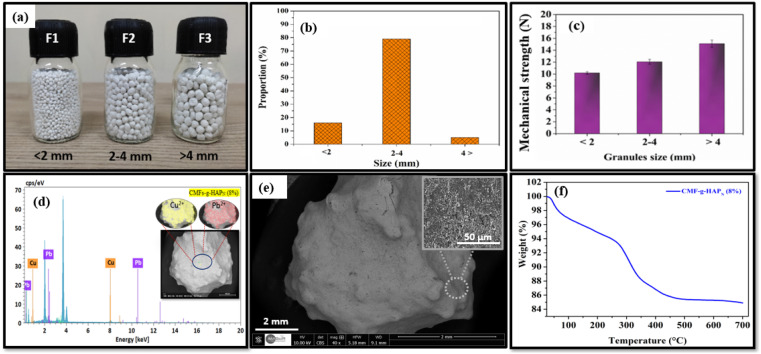
(a) Digital images of CMFs-*g*-HAP_N_ (8%) granules distributed according to their size (mm) and (b) obtained proportion (%) of each size range. (c) Mechanical properties of different resulting fractions. (d) EDX spectrum of prepared CMFs-*g*-HAP_N_ (8%) granules after dynamic adsorption tests. (e) SEM of selected fractions at high magnification. (f) TGA curve of prepared CMFs-*g*-HAP_N_ (8%) granules.

The obtained CMFs-*g*-HAP_N_ (8%) granules as well as their size distribution after granulation are presented in Fig. 1a. The resulting granules of diameter 2–4 mm were the most abundant fraction, with a significantly high proportion up to 80%. Granules <2 mm or >4 mm were classified as “fine” and “course”, respectively, with global average proportion <20%. The mechanical strength of all resulting fractions was evaluated. Granules varied in size between 2 mm and 4 mm and had good mechanical strength up to 12 N (Fig. 1c), which was likely due to the high cohesive force generated from the small intraparticle space in CMFs-*g*-HAP_N_ (8%) material during granulation. Preliminary adsorption tests conducted on the removal of Pb(ii) and Cu(ii) ions using different fractions (*F*1, *F*2, *F*3) demonstrated that granules of size 2 mm to 4 mm exhibited significantly superior adsorption performance compared with those of the other fractions. The effectiveness of adsorption for Cu(ii) and Pb(ii) ions using *F*2 granules was confirmed through energy-dispersive X-ray (EDX) spectroscopy (Fig. 1d). Throughout the continuous adsorption process, distinct peaks specific to lead and copper were detected. Mapping analysis revealed a homogeneous distribution of both ions on the surface of CMFs-*g*-HAP_N_ (8%) granules. This observation provided further support for the successful adsorption of both metals by CMFs-*g*-HAPN (8%) granules. Importantly, *F*3 showed the best properties in terms of mechanical strength, but its application for contaminant removal has limitations such as low adsorption performance and release of some material during adsorption. Therefore, we suggest that the fraction *F*2 had great potential for further optimization of adsorption using a fixed-bed column. On the other hand, SEM was used to evaluate the surface morphology of selected granules (*F*2) (Fig. 1e). Scanning electron micrographs showed the surfaces of granules at high magnification: they were rough. These observations suggested that the irregular morphology of granules could enhance metal adsorption in different portions of these materials due to an increase in surface area. Furthermore, the thermal stability of the selected fraction was also investigated by TGA under air employing a heating rate of 10 °C min^−1^ between 25 and 700 °C. Approximately 3% weight loss was recorded in the range 40–100 °C (Fig. 1e), which could be attributed to the elimination of water molecules. Rapid weight loss at approximately 100–500 °C was attributed to the degradation of organic matter (*e.g.*, cellulose). However, below 40 °C (which is often the maximum operating temperature for fixed-bed adsorption systems^28^), significant weight loss was not observed. This observation indicated that our granules were relatively stable and could not be degraded at natural temperatures.

### 3.2. Study on fixed-bed columns

Dynamic adsorption studies based on the DD were conducted to evaluate the effect of the pre-selected three independent variables on the adsorption capacity of Pb(ii) and Cu(ii) onto CMFs-*g*-HAP_N_ (8%) granules. The fixed-bed length (*X*_1_), effluent flow rate (*X*_2_), and initial concentration of metal (*X*_3_) were chosen as the independent variables. The results obtained for 15 experiments, as well as the responses predicted by Design Expert software and using RSM-DD, are summarized in Table S1, ESI.† The quadratic polynomial model generated by the software was applied to highlight the interaction between independent and dependent variables. The predicted regression model for the two responses (*i.e.*, Pb(ii) adsorption capacity (*Ŷ*_1_, mg g^−1^) and Cu(ii) adsorption capacity (*Ŷ*_2_, mg g^−1^)) can be expressed in eqn (8) and (9):

8 *Ŷ*_1_ = 30.353 + 10.961*X*_1_ − 18.105*X*_2_ + 0.960*X*_3_ + 4.407*X*_1_^2^ − 0.828*X*_2_^2^ −10.721*X*_3_^2^ − 14.572*X*_1_*X*_2_ − 2.852*X*_1_*X*_3_ − 16.475*X*_2_*X*_3_

9 *Ŷ*_2_ = 16.63 + 4.315*X*_1_ − 13.624*X*_2_ + 1.7*X*_3_ + 0.855*X*_1_^2^ + 2.896*X*_2_^2^− 3.845*X*_3_^2^ − 9.967*X*_1_*X*_2_ − 0.913*X*_1_*X*_3_ − 8*X*_2_*X*_3_

Eqn (8) and (9) describe the effect of independent parameters on the adsorption of Pb(ii) and Cu(ii) ions by CMFs-*g*-HAP_N_ (8%) granules. The significance and adequacy of the models were further justified through ANOVA (Table 2). The significance of the model was checked by its *P*-value and *F*-value, whereby *P* <5% was considered significant.^29^

**Table 2:** Analysis of variance (ANOVA) of the adsorption capacity of Pb(ii) and Cu(ii) (mg g^−1^)

Metal	Source	Sum of squares	*D*_f_	Mean square	*F*-Value	*P*-Value (%)
Pb(ii)	Regression	2357.64	9	261.96	23.006	0.150
	Residual	56.935	5	11.386		
	Lack of fit	44.470	3	14.823	2.379	31
	Pure error	12.460	2	6.230		
Cu(ii)	Regression	1007.480	9	111.942	54.339	0.018
	Residual	10.300	5	2.060		
	Lack of fit	7.779	3	2.593	2.0576	34.44
	Pure error	2.520	2	1.260		

**Fig. 2:**
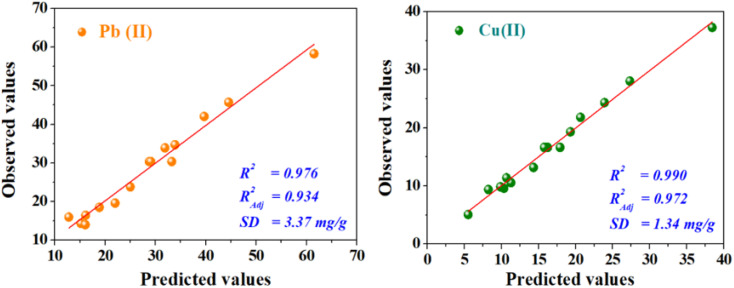
Predicted *vs.* actual values of adsorption capacities of Pb(ii) and Cu(ii) (mg g^−1^).

According to ANOVA (Table 2), a higher value of *F* (23.00 and 54.33 for Pb(ii) and Cu(ii) ions, respectively) showed that most of the variables in the response could be explained by the regression equation, and probability (*P*) values <5% were considered significant. The results compiled by ANOVA predicted adequate representation of the actual relationship between the response (*i.e.*, adsorption capacity) and independent variables (fixed-bed length, effluent flow rate, initial concentration of metal) by a second-order polynomial model. Furthermore, the non-significant value of lack of fit (>5 for both metals) indicated that the quadratic model was valid for the present study. Furthermore, the adjusted values of *R*_adj_^2^ for both metals (0.934 and 0.972 for Pb(ii) and Cu(ii), respectively) were high and of the same magnitude as the predicted values of 0.976 and 0.990, respectively, for Pb(ii) and Cu(ii) (Fig. 2). The difference between the adjusted and predicted values of *R*^2^ was <0.05, which also suggested good agreement between the experimental response and predicted response.

Furthermore, the significance of all linear terms (*X*_1_, *X*_2_, and *X*_3_), quadratic terms (*X*_1_^2^, *X*_2_^2^, and *X*_3_^2^) and interactive terms (*X*_1_*X*_2_, *X*_1_*X*_3_, and *X*_2_*X*_3_) on variations in response was also evaluated by *P*-values (Table S2†). For removal of both metals, the fixed-bed length (*X*_1_), effluent flow rate (*X*_2_), the quadratic term (*X*_3_^2^) as well as the interactive terms (*X*_1_*X*_2_ and *X*_2_*X*_3_), had *P* < 5% and considered to be potentially significant coefficients.^10^ Statistical analyses of the regression coefficients (as listed in Table S2, ESI†) suggested that the fixed-bed length (*X*_1_) had a positive effect, whereas the linear term of effluent flow rate (*X*_2_), the quadratic term (*X*_3_^2^), and the interactive terms (*X*_1_*X*_2_ and *X*_2_*X*_3_) had a negative influence on the adsorption capacity of both metals.

**Fig. 3:**
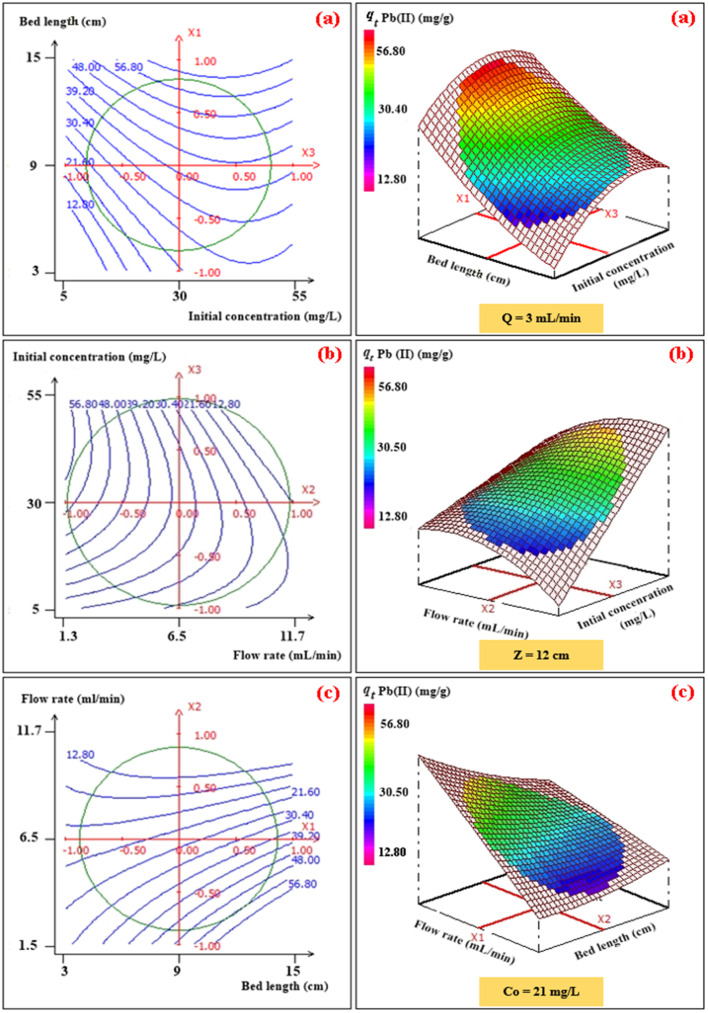
2D–3D surface plots of the effect of bed length and initial concentration (a), flow rate and initial concentration (b), and flow rate and bed length (c) on the adsorption capacity (mg g^−1^) of Pb(ii).

**Fig. 4:**
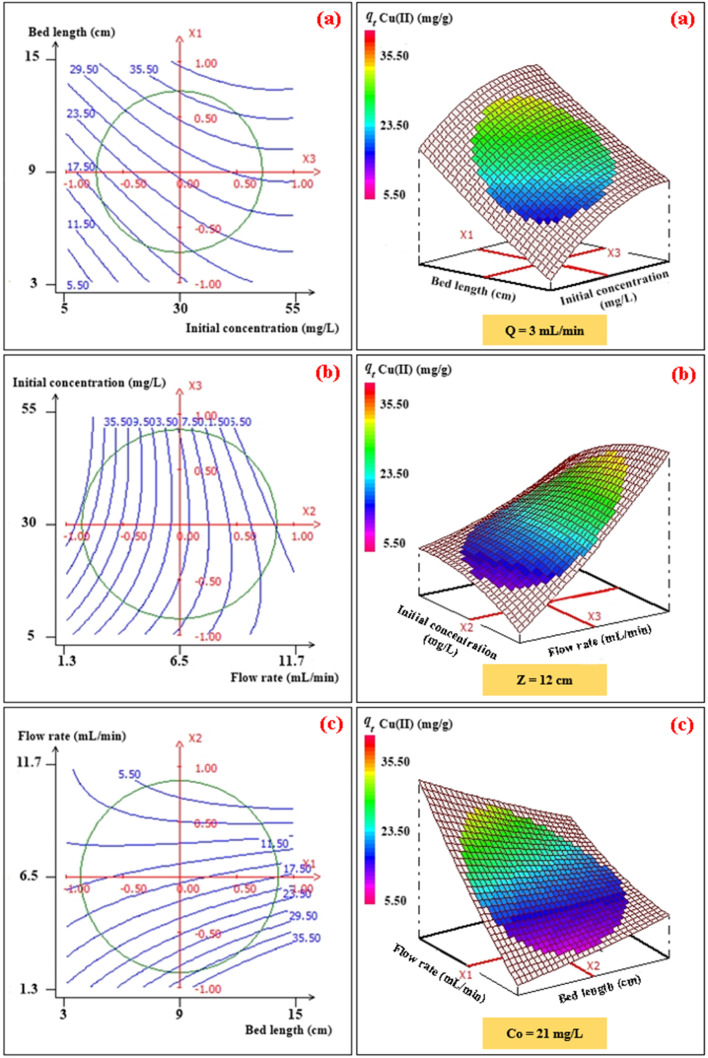
2D–3D surface plots of the effect of bed length and initial concentration (a), flow rate and initial concentration (b), and flow rate and bed length (c) on the adsorption capacity (mg g^−1^) of Cu(ii).

To investigate the interaction among the different independent variables and their corresponding effects on the variation in response (adsorption capacities of Pb(ii) and Cu(ii)), 2D and 3D plots were drawn (Fig. 3 and 4). These plots can be helpful in understanding the main and interaction effects of the independent variables on the response.

#### 3.2.1. Effects of the bed height and initial concentration

The combined effect of bed height (from 3 to 15 cm) and initial concentration (from 10 mg L^−1^ to 50 mg L^−1^) on the adsorption capacity of Pb(ii) and Cu(ii) at a volumetric constant flow rate (3 mL min^−1^) are shown in Fig. 3a and 4a. The response function (*i.e.*, the adsorption capacity of Pb(ii) and Cu(ii)) increased with an increasing initial concentration of ions as well as the bed height. This trend could be explained by the fact that increasing the bed height results in an increased availability of active sites for adsorption and a higher influent concentration provides a higher driving force for transfer to overcome mass-transfer resistance.^30^

#### 3.2.2. Effects of the volumetric flow rate and initial concentration

The combined effect of the initial concentration of the metal and flow rate on adsorption were investigated by varying the flow rate from 2 to 11 mL min^−1^ and the initial concentration from 10 to 50 mg L^−1^, while the fixed bed height was kept constant (12 cm) (Fig. 3b and 4b). The dynamic adsorption capacity of both metals decreased rapidly with an increase in the flow rate for all experimental studies. At a higher volumetric flow rate, the residence time of the ion solution in the fixed bed decreased. Hence, the metal ions did not have sufficient time to catch the active sites on the granule surface or diffuse into the pores of the CMFs-*g*-HAP_N_ (8%) adsorbent, thereby leaving the column before equilibrium could occur.

#### 3.2.3. Effects of the fixed-bed height and volumetric flow

The influence of the fixed-bed height (from 3 cm to 15 cm) and volumetric flow rate (from 2 mg L^−1^ to 11 mg L^−1^) on dynamic adsorption of Pb(ii) and Cu(ii) using CMFs-*g*-HAP_N_ (8%) granules were also investigated (Fig. 3c and 4c). The interactive effect between the flow rate and bed height had a significant impact on the adsorption capacity. The equilibrium adsorption capacity increased significantly from 29.54 to 59.59 mg g^−1^ in the removal of Pb(ii), and from 16.04 to 36.51 mg g^−1^ in the removal of Cu(ii), when the fixed-bed height increased from 5 to 13 cm and at a low flow rate (3 mL min^−1^) (Fig. 3c and 4c). This observation may have been due to the increase in bed depth, which led to an increase in the number of available active sites on the surface of granules. Also, using a slow flow rate provided more contact time between CMFs-*g*-HAP_N_ (8%) granules and metal ions, which led to significant mass transfer through the adsorbent bed. Thus, an increase in the adsorption capacity of the system was observed.

#### 3.2.4. Optimal conditions for continuous adsorption of Pb(ii) and Cu(ii) ions

The “desirability” function in Nemrodw software offers multiple scenarios of optimal values for each factor in accordance with the experimental results. Hence, the optimal response appropriate for the goal of the study can be determined. From multiple responses, the best scenarios selected, as well as the obtained experimental results carried out under these conditions, are summarized in Table 3. Based on the results obtained, three scenarios could be considered to be optimal conditions with different desirability values ranging from 92 to 97%. We concluded that RSM-DD could allow for a better and more purposeful optimization of these studies.

**Table 3:** Predicted adsorption capacities for Pb(ii) and Cu(ii) ions under optimal conditions

Scenario	Factor	Coded value	Real value	Predicted response	Desirability
1	*Z* (cm)	0.720	13	*q*(Pb^2+^)_total_ = 59.59 ± 3.37 mg g^−1^	97%
	*C*_0_ (mg L^−1^)	0.000	30	*q*(Cu^2+^)_total_ = 35.66 ± 1.34 mg g^−1^	
	*Q* (mL min^−1^)	−0.680	3		
2	*Z* (cm)	0.480	12	*q*(Pb^2+^)_total_ = 57.30 ± 3.37 mg g^−1^	93%
	*C*_0_ (mg L^−1^)	0.480	42		
	*Q* (mL min^−1^)	−0.720	2.8	*q*(Cu^2+^)_total_ = 36.14 ± 1.34 mg g^−1^	
3	*Z* (cm)	0.320	11	*q*(Pb^2+^)_total_ = 56.46 ± 3.37 mg g^−1^	92%
	*C*_0_ (mg L^−1^)	0.360	39	*q*(Cu^2+^)_total_ = 36.18 ± 1.34 mg g^−1^	
	*Q* (mL min^−1^)	−0.840	2.1		

#### 3.2.5. Parameters of continuous adsorption

**Fig. 5:**
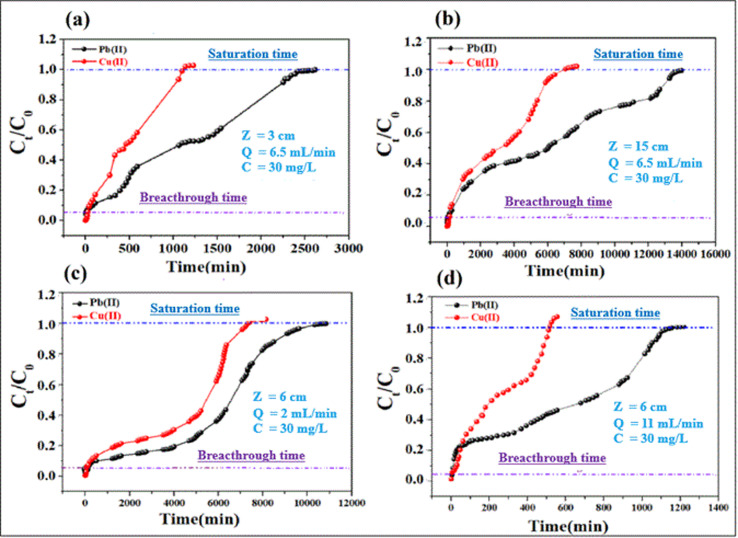
Effect of bed height (a and b) and flow rate (c and d) on the breakthrough profiles for adsorption of Cu(ii) and Pb(ii).

Adsorption of lead and copper by CMFs-g-HAP_N_ (8%) granules was presented in the form of breakthrough curves. In these curves, the concentration ratio *C*_*t*_/*C*_0_ was plotted against time, and the calculated parameters are summarized in Table S3 (ESI).† Also, Fig. 5a and b shows the breakthrough profile of lead and copper adsorption for different bed heights at a given flow rate and concentration. Uptake of Pb(ii) and Cu(ii) increased with an increase in the bed height from 3 to 15 cm. This was reflected in the breakthrough curve with a bed height of 3 cm; granules became saturated early compared with that using other bed heights. The increase in the adsorption capacity of metal ions with an increase in bed height in the column was due to the increase in the number of active sites of the adsorbent, which provided more binding sites for adsorption. The breakthrough time (*t*_p_) for both metal ions also increased with an increase in bed height. Nevertheless, different results were obtained when the effect of flow rate was studied. This can be seen in Fig. 5c and d, where the uptake of Pb(ii) and Cu(ii) decreased with an increase in flow rate from 2 to 11 mL min^−1^. Granules became saturated early at a higher flow rate (11 mL min^−1^). The breakthrough capacity of the granules decreased with the flow rate, which is in agreement with the literature.^31^ At higher flow rates, the residence time of ions in the fixed bed decreases, thereby reducing the contact time between the active sites of the adsorbent and metal ions.

### 3.3. Dynamic modeling of breakthrough curves

**Fig. 6:**
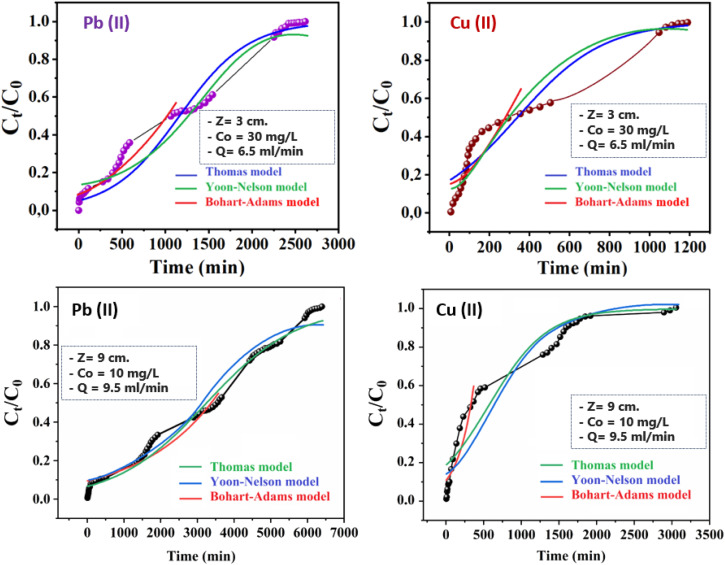
Comparison of experimental and predicted breakthrough curves obtained at various conditions according to Yoon–Nelson, Thomas, and Bohart–Adams models.

Thomas, Yoon–Nelson, and Adams–Bohart mathematical models were implemented to fit the breakthrough curves to describe the dynamic process in the column as well as to determine the corresponding kinetic parameters (Fig. 6).

#### 3.3.1. Evaluation of the parameters of the Adams–Bohart model

The experimental data of the breakthrough curves for dynamic adsorption of Cu(ii) and Pb(ii) ions in a fixed bed were fitted to the Adams–Bohart model at a breakthrough point of 5%. The calculated model parameters along with the correlation coefficient (*R*^2^) values are listed in Table 4.

**Table 4:** Parameters of the Adams–Bohart model by nonlinear regression for the adsorption of Cu(ii) and Pb(ii) ions by CMFs-*g*-HAP_N_(8%) granules

Experimental conditions			Cu(ii)			Pb(ii)		
			Adams–Bohart parameters			Adams–Bohart parameters		
*Z* (cm)	*C*_0_ (mg L^−1^)	*Q* (mL min^−1^)	*K*_AB_ (mL mg^−1^ min^−1^) × 10^−4^	*N*_0_ (mg L^−1^)	*R* ^2^	*K*_AB_ (mL mg^−1^ min^−1^) × 10^−4^	*N*_0_ (mg L^−1^)	*R* ^2^
15	30	6.5	0.00053 × 10^−1^	44.04	0.89	0.0002	13.80	0.87
3	30	6.5	0.0037	47.62	0.66	0.0021	81.53	0.72
6	30	2	0.00031 × 10^−1^	71.22	0.89	0.0003 × 10^−1^	88.95	0.93
6	30	11	0.0026	8.03	0.77	0.0059	3.14	0.80
12	50	8	0.0011	9.52	0.78	0.0022	9.53	0.69
9	10	9.5	0.0033	21.82	0.93	0.0040	61.90	0.96

The kinetic constant of the model (*k*_AB_) increased with decreasing bed height and initial concentration for both metal ions, but decreased with increasing flow rate (Table 4). These data implied that the reaction kinetics were strongly affected by external mass transfer in the initial part of adsorption in the column.^32^ However, the adsorption capacity of granules (*N*_0_) decreased significantly with increasing volumetric flow, which confirmed the antagonist effect of the flow rate on adsorption of Pb(ii) and Cu(ii) using CMFs-*g*-HAP_N_ (8%) granules. A similar trend was observed for adsorption of Pb(ii) onto nanostructured γ-alumina in a fixed bed by increasing the flow rate from 3 to 9 mL min^−1^: *N*_0_ decreased significantly.^33^ Moreover, the predicted curves for both ions were compared with the corresponding experimental curves in Fig. 6 under different experimental conditions. There was good agreement between experimental data and predicted values, suggesting that the Adams–Bohart model would be valid for a relative concentration region up to 0.5. Large discrepancies were found between experimental and predicted curves above this level for the dynamic adsorption of Pb(ii) and Cu(ii) using CMFs-*g*-HAP_N_ (8%) granules (this part of predicted curve is not presented in Fig. 6). The Adams–Bohart model provided a straightforward and inclusive method for conducting and assessing sorption-column tests, but its applicability was limited to the range of conditions employed.

#### 3.3.2. Evaluation of parameters of the Thomas model

To determine the maximum adsorption capacity of the adsorbent (*q*_Th_) and kinetic coefficient (*k*_Th_) in the Thomas model, experimental data were fitted into the eqn (5). The calculated parameters and regression analysis coefficients are presented in Table 5. According to Table 5, as the bed height increased from 3 to 15 cm, the values of adsorption capacity *q*_Th_ (mg g^−1^) and kinetic coefficient of the model (*k*_Th_) increased and decreased, respectively. Simultaneously, as the flow rate increased from 2 to 11 mL min^−1^, the values of *q*_Th_ (mg g^−1^) decreased but the values of (*k*_Th_) increased, which could be explained by the unavailability of active sites for the reaction. Fig. 6 also presents the predicted curves according to the Thomas model. It is clear from Fig. 6 and Table 5 that there was good agreement between the experimental and predicted normalized concentration values at experimental conditions. The Thomas model was suitable for adsorption processes in which the external diffusion and internal diffusion were not the limiting step. A similar trend was observed by Kavianinia *et al.* during adsorption studies of Cu(ii) onto modified chitosan hydrogel.^34^

**Table 5:** Parameters of the Thomas model by nonlinear regression analysis for the adsorption of Cu(ii) and Pb(ii) ions by CMFs-*g*-HAP_N_ (8%) granules

Experimental conditions			Cu(ii)				Pb(ii)			
			Thomas parameters				Thomas parameters			
*Z* (cm)	*C*_0_ (mg L^−1^)	*Q* (mL min^−1^)	*k*_Th_ (mL min^−1^ mg^−1^) × 10^−4^	*q*_Th_ (mg g^−1^)	*k*_Th_ (mL min^−1^ mg^−1^) × 10^−4^	*R* ^2^	*q*_Th_ (mg g^−1^)	*k*_Th_ (mL min^−1^ mg^−1^) × 10^−4^	*q*_Th_ (mg g^−1^)	*R* ^2^
15	30	6.5	0.0007 × 10^−1^	32.3	0.97	0.97	0.0003 × 10^−1^	58.3	0.95	0.95
3	30	6.5	0.0046	3.39	0.93	0.93	0.0033	9.12	0.99	0.99
6	30	2	0.0005 × 10^−1^	45.3	0.95	0.95	0.0007 × 10^−1^	61.6	0.94	0.94
6	30	11	0.0036	5.55	0.96	0.96	0.0047	3.97	0.94	0.94
12	50	8	0.0033 × 10^−1^	6.31	0.95	0.95	0.0026	7.38	0.94	0.94
9	10	9.5	0.0013 × 10^−1^	17.3	0.98	0.98	0.0019	42	0.96	0.96

#### 3.3.3. Evaluation of parameters of the Yoon–Nelson model

A simple theoretical model developed by Yoon–Nelson was also applied to investigate the breakthrough behaviour for adsorption of Pb(ii) and Cu(ii) ions on CMFs-*g*-HAP_N_ (8%) granules. The values of the Yoon–Nelson constant (*K*_YN_) and *τ* for 50% adsorbate breakthrough time were calculated from the plot of ln[*C*_*t*_/(*C*_o_ − *C*_*t*_)] *versus* time at different flow rates, different initial cation concentrations, and at different bed heights. The values of all calculated parameters are presented in Table 6. The results of the fitting revealed that the rate constant (*k*_YN_) increased as much as flow rate increased. Also, the time required for 50% adsorbate breakthrough (*τ*) decreased with an increasing flow rate due to the lower residence time of cations in the granule bed. Comparison of the experimental points and predicted curves according to the Yoon–Nelson model are also shown in Fig. 6. From experimental results and data regression (*R*^2^ values from 0.93 to 0.99 for both metal ions), the Yoon–Nelson model provided a good correlation. Consequently, nonlinear regression analysis of experimental data demonstrated that Thomas and Yoon–Nelson models were appropriate to explain the breakthrough curve, whereas the Adams–Bohart was applicable only to predict the initial part of the dynamic process.

**Table 6:** Parameters of the Yoon–Nelson model using nonlinear regression analysis for adsorption of Pb(ii) and Cu(ii) onto CMFs-*g*-HAP_N_ (8%) granules under various operating conditions

Experimental conditions			Cu(ii)			Pb(ii)		
			Yoon–Nelson parameters			Yoon–Nelson parameters		
*Z* (cm)	*C*_0_ (mg L^−1^)	*Q* (mL min^−1^)	*K*_YN_ (min^−1^) × 10^−4^	*τ* (min)	*R* ^2^	*K*_YN_ (min^−1^) × 10^−4^	*τ* (min)	*R* ^2^
15	30	6.5	0.0007 × 10^−1^	3256	0.97	0.0003 × 10^−1^	5830	0.95
3	30	6.5	0.0046 × 10^−1^	339	0.93	0.0033	912	0.99
6	30	2	0.0005 × 10^−1^	4536	0.95	0.0007 × 10^−1^	6196	0.94
6	30	11	0.0036	555	0.96	0.0047	397	0.94
12	50	8	0.0033	631	0.95	0.0026 × 10^−1^	738	0.94
9	10	9.5	0.0013 × 10^−1^	1739	0.98	0.0019 × 10^−1^	4204	0.96

### 3.4. Desorption and column regeneration

**Fig. 7:**
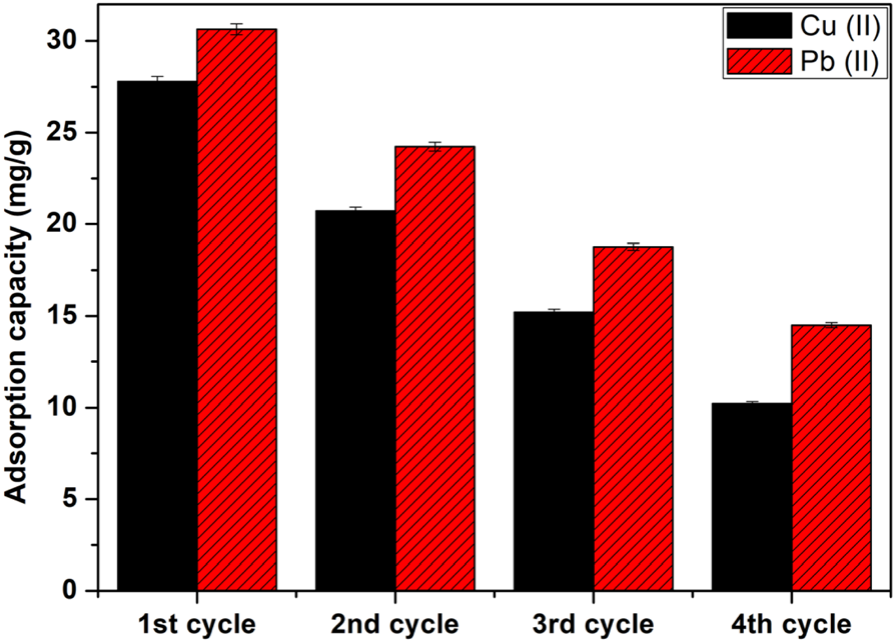
Adsorption–desorption cycles of Cu(ii) and Pb(ii) onto CMFs-*g*-HAP_N_ (8%) granules.

Study of the regeneration and reuse of CMFs-*g*-HAP_N_ (8%) adsorbent in a continuous process was achieved to determine the lifespan of granules as well as their applicability in water-treatment processes, particularly from economic and environmental viewpoints. In this step, chelating agents or acids as desorption solutions are used generally.^35,36^ Our previous batch studies showed that EDTA-Na_2_ (0.001 M) appeared to be the best eluant capable of desorbing >60% for Pb(ii) and 70% for Cu(ii) compared with all eluting agents tested for desorption due to its strong ability to form a stable complex with Pb(ii) and Cu(ii) ions.^37^ Therefore, in the present study, EDTA-Na_2_ was used as an “ideal” eluent for desorption of Pb(ii) and Cu(ii) ions. After adsorption, granules were rinsed thoroughly with distilled water to remove non-adsorbed metal ions. Then, EDTA-Na_2_ (0.001 M) was passed through a column at a flow rate of 2 mL min^−1^ to regenerate CMFs-*g*-HAP_N_ (8%) granules. Then, the regenerated granules were used as a recyclable adsorbent for the removal of metal ions in numerous recycling cycles. As seen in Fig. 7, CMFs-*g*-HAP_N_ (8%) granules could be reused consecutively for recovery of metal ions with a gradual drop in their uptake ability after four successive adsorption–desorption cycles. This decrease could be attributed to the loss of binding sites after each adsorption–desorption step.^38^

### 3.5. Adsorption mechanism

**Scheme 1:**
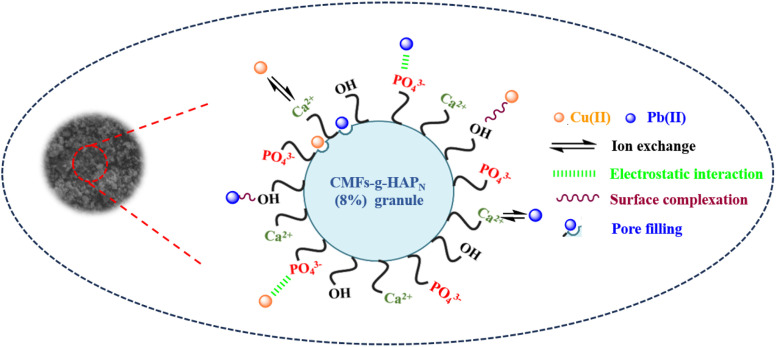
Postulated mechanism of adsorption of Pb(ii) and Cu(ii) onto CMFs-*g*-HAP_N_ (8%) granules.

The adsorption mechanism of a contaminant on an adsorbent is dependent upon several factors, including the properties of the adsorbent, the nature of the adsorbate, and the potential interactions between the adsorbate and adsorbent. The experimental studies stated above as well as the desorption process emphasized the importance of surface complexation and electrostatic interactions, which manifested primarily as physical mechanisms. Through surface complexation, metal ions form coordination complexes with available hydroxyl and phosphate groups on the surfaces of granules. This intricate coordination enhances the adsorption capacity and stabilizes the metal ions on the surface. Electrostatic interactions involve the attraction and binding of positively charged metal ions by negatively charged phosphate and hydroxyl groups, thereby promoting their adherence. Furthermore, the rough and porous surface of CMFs-*g*-HAPN (8%) granules observed in SEM images, along with the significant surface area and abundance of mesopores identified by BET analysis in our previous work,^9^ suggest a possible pore-filling mechanism. This implies that Pb(ii) and Cu(ii) ions may infiltrate and occupy these pores, potentially contributing to the overall adsorption process. In addition, the Ca^2+^ concentration in the bulk solution was analyzed before and after the adsorption of Pb(ii) and Cu(ii) onto CMFs-*g*-HAPN (8%) granules. This particular test revealed that these two metals underwent partial exchange with Ca^2+^ during adsorption. On the other hand, the competitive interaction between Pb(ii) and Cu(ii) for adsorption sites introduces complexity, resulting in varying adsorption capacities. This competition may favor the preferential adsorption of one metal ion over the other. Specifically, Pb(ii) engages in chemisorption, which involves the sharing of electrons with surface hydroxyl groups, to form stable Pb–OH complexes on the surface. Cu(ii) can also interact with surface hydroxyl groups, but may exhibit a weaker affinity compared with that of Pb(ii). This affinity could be attributed to the higher Pauling electronegativity of Pb (2.33) compared with that of Cu (1.90), which favors electrostatic complexation and interactions between the inner sphere and surface.^39–41^ Based on these findings, a comprehensive scheme was proposed (Scheme 1) to elucidate the intricate mechanisms governing the adsorption of Pb(ii) and Cu(ii) onto the CMFs-*g*-HAP_N_ (8%) column.

### 3.6. Comparison with other adsorbents

According to the literature, several adsorbents have been investigated for the removal of lead and copper from aqueous solutions using fixed-bed columns. The competitiveness of our adsorbent was evaluated in comparison with that of other reported adsorbents (Table 7). As indicated in Table 7, the suggested adsorbent demonstrated comparable adsorption capacity and a strong attraction towards Pb(ii) and Cu(ii) in comparison with that of other adsorbents. Moreover, the developed adsorbent was novel, competitive, effective, practical, and inexpensive. These observations suggest that the prepared CMFs-*g*-HAP_N_ (8%) granules could be efficient adsorbents because of their simple preparation, easy recovery, and reusability, which is favorable for the treatment of industrial effluents containing heavy metals.

**Table 7:** Adsorption capacity of Pb(ii) and Cu(ii) ions using various adsorbents in column mode

Metal	Adsorbents	Adsorption capacity (mg g^−1^)	Ref.
Pb(ii)	Chaff	6.72	42
	Coconut shell	2.01	43
	Mushroom	90	44
	CMFs-*g*-HAP_N_ (8%)	59.59	Present work
	Sago waste	46.6	45
	Papaya wood	17.4	46
	PAAC nanocomposite	36.20	47
Cu(ii)	Chaff	1.98	42
	Almond shell	2.39	48
	Palm oil boiler mill fly ash	21.93	49
	CMFs-*g*-HAP_N_ (8%)	35.66	Present work
	Polyaniline-coated sawdust	58.23	50
	Magnetized sawdust (Fe_3_O_4_-SD)	43.45	51

## 4. Conclusions

We investigated the performance of CMFs-*g*-HAP_N_ (8%) granules for simultaneous removal of Pb(ii) and Cu(ii) in a fixed-bed system. RSM application proved to be effective in describing, modeling, and optimizing the effects of operational parameters (initial concentration of metal ion, flow rate, and bed length) on the adsorption capacity. Removal efficiency increased with bed length and the initial concentration of Pb(ii) and Cu(ii), but it decreased with the flow rate. The optimal conditions for removal of 59.59 and 35.66 mg g^−1^ of Pb(ii) and Cu(ii), respectively, were predicted to be a bed length of 13 cm of CMFs-*g*-HAP_N_ (8%) granules, an initial concentration of metal of 30 mg L^−1^, and a flow rate of 3 mL min^−1^. The Adams–Bohart, Yoon–Nelson, and Thomas models were applied to experimental data for a fixed bed to predict breakthrough curves and derive kinetic parameters of the column. The CMFs-*g*-HAP_N_ (8%) adsorbent exhibited higher affinity to Pb(ii) ions than for Cu(ii) ions. Given its high performance and recyclability, the CMFs-*g*-HAP_N_ (8%) adsorbent could be a commercial adsorbent used widely for the removal of heavy metals from wastewater.

## Conflicts of interest

The authors declare that there is no conflicts of interest regarding publication of this manuscript.

## Supplementary Material

RA-013-D3RA04974D-s001
