# Supplementary material for: Fixed-bed adsorption of Pb(ii) and Cu(ii) from multi-metal aqueous systems onto cellulose-g-hydroxyapatite granules: optimization using response surface methodology
Source: RSC Adv. 2023 Nov 1;13(45):31935–47. doi: 10.1039/d3ra04974d (PMC10618904; doi:10.1039/d3ra04974d)
Supplement: RA-013-D3RA04974D-s001 [file RA-013-D3RA04974D-s001.pdf]

Electronic Supplementary Material (ESI) for RSC Advances.
This journal is © The Royal Society of Chemistry 2023

Electronic Supporting Information (ESI)

Fixed bed adsorption of Pb (II) and Cu (II) from multi-metal aqueous systems onto Cellulose-*g*-Hydroxyapatite granulates: Optimization using response surface methodology.

Salah Eddine Marrane[a], Karim Dänoun*[bc], Youness Essamlali[bc], Soumia Aboulhrouz[bc], Said Sair[bc], Othmane Amadine[bc], Ilham Jioui[bc], Abdallah Rhihil[a], Mohamed Zahouily*[abc]

[a]*Laboratory of Materials, Catalysis & Valorization of Natural Resources, Hassan II University, FST-Mohammedia, Morocco.*
[b]*Moroccan Foundation for Advanced Science, Innovation and Research (MAScIR),Benguerir, Morocco.*
[c]*Mohammed VI Polytechnic University, Lot 660-Hay Moulay Rachid, Benguerir, Morocco.*

E-mail: m.zahouily@mascir.ma; karim.DANOUN@um6p.ma.

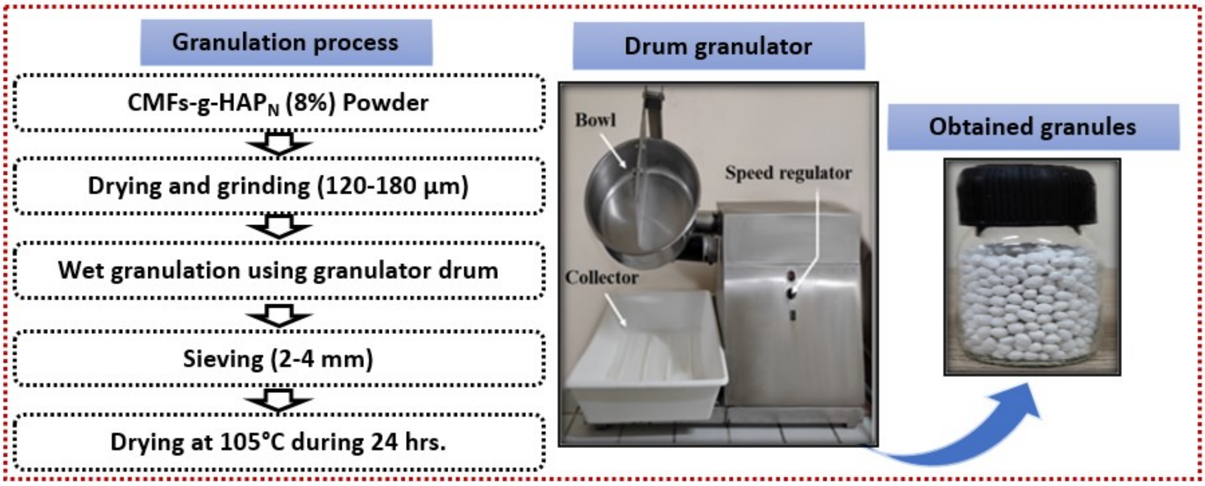

Scheme S1: the preparation steps of CMFs-g-HAP_N (8%) granulates.

Table S1: Design matrix for three coded variables together with the actual and predicted responses.

Runs	Coded values of the variables						
	X_1	X_2	X_3	$Y_{1(Pb^{2+})}$ (mg/g)	$\hat{Y}_{1(Pb^{2+})}$ (mg/g)	$Y_{2(Cu^{2+})}$ (mg/g)	$\hat{Y}_{2(Cu^{2+})}$ (mg/g)
**1**	15	6.5	30	44.53	45.72	20.65	21.80
**2**	3	6.5	30	24.99	23.80	14.32	13.17
**3**	12	11.0	30	15.19	14.34	5.5	5.07
**4**	6	2.0	30	33.87	34.72	23.9	24.33
**5**	12	2.0	30	61.5	58.29	38.43	37.27
**6**	6	11.0	30	12.78	15.99	8.22	9.38
**7**	12	8.0	50	18.85	18.51	11.28	10.56
**8**	6	5.0	10	16.11	16.45	10.67	11.39
**9**	12	5.0	10	31.88	33.90	19.3	19.31
**10**	9	9.5	10	21.97	19.61	10.33	9.61
**11**	6	8.0	50	16.05	14.03	9.86	9.85
**12**	9	3.5	50	39.67	42.03	27.32	28.05
**13**	9	6.5	30	28.76	30.35	16.22	16.63
**14**	9	6.5	30	29.07	30.35	15.77	16.63
**15**	9	6.5	30	33.23	30.35	17.9	16.63

Table S2: Estimates and statistics of the coefficients for Pb(II) and Cu(II) adsorption capacities ($mg.g^{-1}$).

	Pb(II) adsorption capacity			Cu(II) adsorption capacity		
Terms	Coefficient	t-Value	P-Value %	Coefficient	t-Value	P-Value %
Constant	30.353	15.58	< 0.01 ***	16.630	20.07	< 0.01 ***
X_1	10.961	6.50	0.129 **	4.315	6.01	0.183 **
X_2	-18.105	-10.72	0.0122 ***	-13.624	-18.97	< 0.01 ***
X_3	0.960	0.56	60.1	1.700	2.32	6.8
X_1X_1	4.407	1.43	21.2	0.855	0.65	54.3
X_2X_2	-0.828	-0.27	79.9	2.896	2.21	7.8
X_3X_3	-10.721	-3.52	1.69 *	-3.845	-2.97	3.12 *
X_1X_2	-14.572	-3.74	1.35 *	-9.967	-6.01	0.183 **
X_1X_3	-2.852	-0.64	54.9	-0.913	-0.48	65.0
X_2X_3	-16.475	-3.70	1.40 *	-8.000	-4.23	0.827 **

Table S3. Packed-bed parameters for adsorption Pb (II) and Cu (II) ions by CMFs-g-HAP_N (8%) fixed bed.

Tests	Operational conditions	Metals	$V_{eff,i}$ (L)	$t_{p,i}$ (min)	$t_{s,i}$ (h)	Q_{tot} of Pb	Q_{tot} of Cu
**1**	15 cm, 30 mg/L ,6.5 mL/min	Pb(II)	91.32	17	233	44.53	20.65
		Cu(II)	44.63	10	114		
**2**	3 cm, 30 mg/L ,6.5 mL/min	Pb(II)	17.15	7	44	24.99	14.32
		Cu(II)	7.69	5	20		
**3**	12 cm, 30 mg/L ,11 mL/min	Pb(II)	29.96	9	45	15.19	5.5
		Cu(II)	9.87	6	15		
**4**	6 cm, 30 mg/L ,2 mL/min	Pb(II)	20.50	67	164	33.87	23.9
		Cu(II)	14.82	44	122		
**5**	12 cm, 30 mg/L , 2 mL/min	Pb(II)	56.23	86	460	61.5	38.43
		Cu(II)	49.25	60	380		
**6**	6 cm, 30 mg/L ,11 mL/min	Pb(II)	13.69	6	20	12.78	8.22
		Cu(II)	11.33	3	17		
**7**	12 cm, 50 mg/L , 8 mL/min	Pb(II)	12.18	47	25	18.85	11.28
		Cu(II)	9.71	35	20		
**8**	6 cm, 10 mg/L ,5 mL/min	Pb(II)	34	29	113	16.11	10.67
		Cu(II)	19.60	12	65		
**9**	12 cm, 10 mg/L ,5 mL/min	Pb(II)	99.89	48	333	31.88	19.3
		Cu(II)	63.89	24	215		
**10**	9 cm, 10 mg/L ,9.5 mL/min	Pb(II)	52.02	26	91	21.97	10.33
		Cu(II)	34.94	16	62		
**11**	6 cm, 50 mg/L ,8 mL/min	Pb(II)	7.64	72	16	16.05	9.86
		Cu(II)	5.51	38	11		
**12**	9 cm, 50 mg/L ,3.5 mL/min	Pb(II)	27.54	122	131	39.67	27.32
		Cu(II)	21.35	88	102		
**13**	9 cm, 30 mg/L ,6.5 mL/min	Pb(II)	31.64	43	81	28.76	16.22
		Cu(II)	21.95	30	56		
**14**	9 cm, 30 mg/L ,6.5 mL/min	Pb(II)	31.12	41	81	29.07	15.77
		Cu(II)	21.90	28	56		
**15**	9 cm, 30 mg/L ,6.5 mL/min	Pb(II)	31.88	39	81	33.23	17.9
		Cu(II)	21.92	27	56		
